# OPLS-Based Multiclass Classification and Data-Driven Interclass Relationship Discovery

**DOI:** 10.1021/acs.jcim.4c01799

**Published:** 2025-02-03

**Authors:** Edvin Forsgren, Benny Björkblom, Johan Trygg, Pär Jonsson

**Affiliations:** † Computational Life Science Cluster (CLiC), Department of Chemistry, Umeå University, SE-901 87 Umeå, Sweden; ‡ Department of Chemistry, Umeå University, SE-901 87 Umeå, Sweden; § Sartorius Corporate Research, SE-903 33 Umeå, Sweden

## Abstract

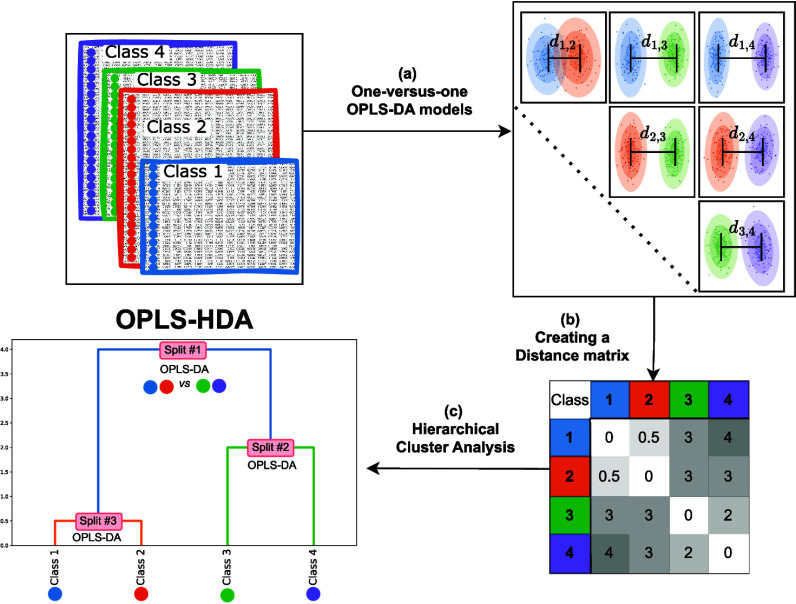

Multiclass data sets and large-scale studies are increasingly common in omics sciences, drug discovery, and clinical research due to advancements in analytical platforms. Efficiently handling these data sets and discerning subtle differences across multiple classes remains a significant challenge. In metabolomics, two-class orthogonal projection to latent structures discriminant analysis (OPLS-DA) models are widely used due to their strong discrimination capabilities and ability to provide interpretable information on class differences. However, these models face challenges in multiclass settings. A common solution is to transform the multiclass comparison into multiple two-class comparisons, which, while more effective than a global multiclass OPLS-DA model, unfortunately results in a manual, time-consuming model-building process with complicated interpretation. Here, we introduce an extension of OPLS-DA for data-driven multiclass classification: orthogonal partial least squares-hierarchical discriminant analysis (OPLS-HDA). OPLS-HDA integrates hierarchical cluster analysis (HCA) with the OPLS-DA framework to create a decision tree, addressing multiclass classification challenges and providing intuitive visualization of interclass relationships. To avoid overfitting and ensure reliable predictions, we use cross-validation during model building. Benchmark results show that OPLS-HDA performs competitively across diverse data sets compared to eight established methods. This method represents a significant advancement, offering a powerful tool to dissect complex multiclass data sets. With its versatility, interpretability, and ease of use, OPLS-HDA is an efficient approach to multiclass data analysis applicable across various fields.

## Introduction

Partial least-squares or projection to latent structures (PLS) regression was first introduced in the 1970s by Herman Wold to handle cases where there are more descriptor variables than observations.^1^ Back then, PLS was built to predict a single response variable. Since then, it has evolved in several steps^2^ with orthogonal PLS (OPLS) being introduced in 2002.^3^ OPLS simplifies interpretation by dividing the variation in descriptor variables into two parts: “predictive” variation related to the response(s) and “orthogonal” variation i.e., not related to the response(s). Regarding predictive power, OPLS and PLS are identical for single response variable models but due to the simplified interpretation, OPLS has gained popularity in fields where understanding the connection between descriptor- and response variables is crucial. Often, the response variable is categorical. Although regression was the original use of both PLS and OPLS, they are now commonly used for classification or discriminant analysis (DA) to distinguish between two or more groups or classes of samples. This type of classification works well for two classes, both in terms of discrimination and interpreting differences between classes. Several methods have been developed for interpreting differences between classes in two-class OPLS-DA models e.g., statistical total correlation spectroscopy (STOCSY),^4^ the S-plot^5^ and selective-ratio.^6^ The latter was initially developed for PLS-DA but later adopted for OPLS-DA.^7^ However, as the number of classes increases, PLS/OPLS-DA suffer in both interpretability and predictive power. The decline in performance is due to the “one-vs-rest” classification approach used in PLS/OPLS-DA models. This setup presents a challenge when one class is positioned between two others, which, combined with the linear nature of PLS/OPLS, results in weak models and complex interpretation.

A common approach to deal with this problem is to make multiple pairwise comparisons, “one-vs-one” models. These models can then be compared using SUS-plots (Shared and Unique Structure).^5^ SUS-plots are especially useful when multiple groups are compared against one control or reference group, such as different genotypes vs wild-type or different treatments vs untreated controls. This manual approach works well when the number of classes is small but quickly becomes challenging to manage as the number of classes increases. Despite these challenges, pairwise OPLS-DA is still widely adopted in omics for interpreting biological differences between different classes. Applications include differentiating brain cancer types,^8^ metabolomics in diet intervention studies,^9^ metabolite distribution patterns,^10^ and gene feature selection with microarray data.^11^ While manually creating multiple “one-vs-one” OPLS-DA models has been proven useful, it is a time-consuming process, both when creating the models and to summarize them into comprehensive results.

To circumvent most of the manual work, the Automatic Hierarchical Model Builder (AHiMBu)^12,13^ was introduced to handle these multiclass cases. And, although more effective than PLS-DA and a manual approach, there is still improvements to be made in terms of flexibility and interpretability.

Here, we introduce a data-driven methodology that combines two proven frameworks hierarchical cluster analysis (HCA) with OPLS-DA, to create OPLS-hierarchical discriminant analysis (OPLS-HDA). OPLS-HDA is developed to handle multiclass data sets where there are different levels of variation to reveal the hierarchical relations between classes while still detailing the discriminatory level. Employing OPLS-DA models as the foundation and a flexible approach to the hierarchical structure, provides OPLS-HDA with significant advantages over existing methods. In addition to increased interpretability and flexibility, we also show that OPLS-HDA has competitive classification abilities compared to other popular methods on a wide range of data sets. In this work, we use OPLS as the framework, though it is important to note that a PLS-based framework would have the same predictive ability.

## Theory

### Orthogonal Projections to Latent Structure Discriminant Analysis—OPLS-DA

OPLS is a supervised multivariate projection method that uses latent variables to establish a linear relationship between the descriptor variables in the predictor matrix (**X**) and a response vector (**y**) or matrix (**Y**) for a specified set of observations. OPLS can be used even when the number of observations is smaller than the number of variables. It can handle correlated variables, noisy variables and missing values. This robust and versatile technique is applicable to a wide range of analytical tasks, including regression^3^ and discrimination,^14^ the latter being facilitated through OPLS-DA. In OPLS-DA, the response matrix (**Y**) is a dummy matrix that contains the information about class membership for each observation. OPLS separates the variation described by the model into two different parts, predictive and orthogonal. The predictive part is the variation in **X** (eq 1) that is used to model the variation in **Y** (eq 2). The orthogonal part contains variation in **X** (eq 1) that is unrelated to the response **Y**. In the OPLS-DA context, the predictive part contains between class variation while the orthogonal part contains within class variation. By dividing the variation into two parts, the interpretation of the model becomes easier.
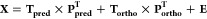
1
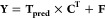
2

### Cross-Validation—CV

By dividing the training set into *k* groups and iterating over the groups, leaving one group out at a time, we can validate our model and get an estimation on how well it will perform on a test set. The cv-predictions i.e., prediction of samples when they are left out of the model denoted as ***ŷ***_cv_, are used to determine the number of components to use in the final model by using the prediction error to decide whether to keep an additional component or not. The ***ŷ***_cv_ and the cv-scores of the samples in the training data set are also useful to understand and interpret the data in a more realistic way than pure predictions of samples that are present in the model and is especially useful if data is scarce.^15^

### Distance Metric

The cv-predictions from two-class OPLS-DA models can be used to estimate the separability between classes. A suitable distance metrics for this is Cohen’s *d*,^16^ defined in eq 3, which takes into account both the distance between classes and variation within them.
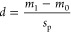
3Here, *m*_0_ and *m*_1_ are the means of the ***ŷ***_cv_ of the two classes and *s*_p_ the pooled standard deviations defined as in eq 4.
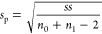
4Here, *ss* is the pooled sum of squares of the two classes and *n* is the number of observations in each class. The distance between the class means represents how well the OPLS-DA model can separate the classes, while the pooled standard deviation is a measure of the variability within the two classes. A model that can clearly distinguish between the two classes will result in a strong and valid model, characterized by predicted responses that are close to the actual targets (0 or 1) and a small pooled standard deviation, leading to a large Cohen’s *d* value. Conversely, in the case of weak models, the predictions for samples from both classes cluster close to the mean value of the *y*-vector. For a balanced data set, this value equals 0.5. This clustering is typically accompanied by a large standard deviation, which indicates poor separation between the classes. Consequently, such a scenario results in a small Cohen’s *d* value.

### Hierarchical Cluster Analysis—HCA

HCA seeks the hierarchy of groups or individual observations based on a symmetrical distance matrix. There are two types of HCA, “bottom-up” (agglomerative) or “top-down” (divisive). For agglomerative, each observation starts as their own cluster and is then grouped successively. For divisive, all observations start in one cluster and are then divided into smaller ones successively. In this paper, we will refer to agglomerative HCA as HCA.

## Methodology

An OPLS-HDA model integrates the global interpretation and visual framework of dendrograms created with HCA with the predictive strength and detailed interpretation benefits of two-class OPLS-DA models. Put simply, an OPLS-HDA model is a top-down decision tree with a two-class OPLS-DA model in each decision node.

**Figure 1:**
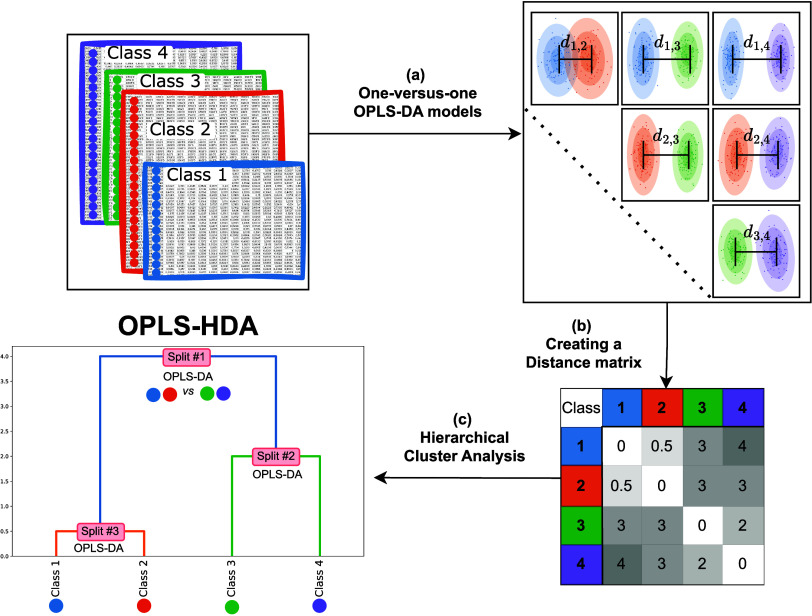
Observations of known classes, characterized by a common set of variables, serve as the starting point. The next step, (a), involves calculating all possible pairwise OPLS-DA models. From these models, Cohen’s *d*s between classes are derived. These distances are compiled into a distance matrix, (b), which then forms the basis for hierarchical clustering analysis with a dendrogram as an end result, (c). The dendrogram reveals the relationships between classes and functions as the basis of a decision tree, with a two-class OPLS-DA model at each split. This dendrogram, in conjunction with OPLS-DA models, constitutes the OPLS-HDA model.

To create the hierarchy of the OPLS-HDA model, the relationships between all classes are mapped. This mapping is achieved by calculating all combinations of one-vs-one OPLS-DA models. Based on these models, the Cohen’s *d* between all the classes is calculated and stored in a distance matrix. This matrix is then used to create a dendrogram that forms the basis of the top-down decision tree populated with two-class OPLS-DA models. This integration creates a model that is interpretable at two levels: the hierarchical level, where the structure of the decision tree reveals the similarities and differences between classes, and the discrimination level, where the OPLS-DA models reveal details regarding how classes or groups of classes are differentiated from one another. An overview is shown in Figure 1 and a detailed description of the setup and the model building is given below.

The method is outlined in Figure 1. All pairwise combinations of classes are compared using OPLS-DA models (Figure 1a). The total number of models calculated in this step is , where *N* is the number of classes. This step is the most time-consuming part of the process, but when automated, still easily doable using a standard computer. The calculated distances are summarized in a symmetrical distance matrix, with each element representing the distance between two classes (Figure 1b). The matrix captures the relational dynamics among all classes. The distance matrix is used to reveal the hierarchical level of the data in a dendrogram using HCA (Figure 1c). The dendrogram is then populated with decision making OPLS-DA models in each split to create the decision tree, i.e., the OPLS-HDA model.

When using the OPLS-HDA model for classification, an observation begins at the top of the decision tree and moves downward based on the predictions of each OPLS-DA model. It ultimately reaches one of the bottom leaves, indicating the class to which the observation belongs.

### Illustrative Comparison of OPLS-HDA and OPLS-DA

To illustrate OPLS-HDA and show its benefits even in simple cases, we compared it to OPLS-DA using the Iris three-class data set.^17^ For simplicity in visualization, we limited the data set to only two variables: petal length and petal width.

**Figure 2:**
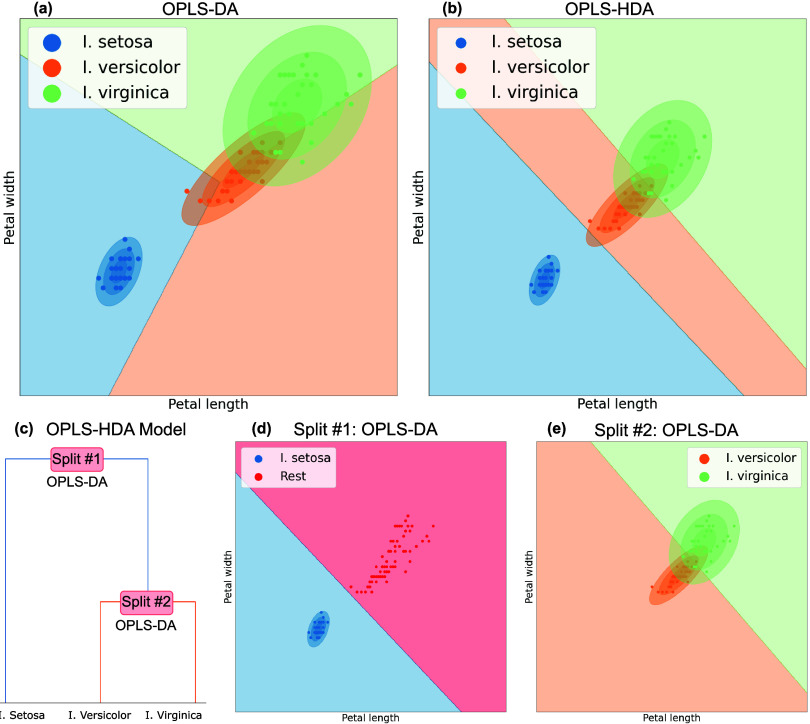
In (a, b) the decision boundaries of an OPLS-DA and an OPLS-HDA model are displayed. The OPLS-DA model has a common center-point leading to a suboptimal decision boundary while OPLS-HDA creates a suitable boundary. In (c), the dendrogram on which the OPLS-HDA model is based. In each split of the dendrogram, a two-class OPLS-DA model is created. In (d), the decision boundary of the model in split #1 is shown, and in (e), the decision boundary of the model in split #2.

OPLS-DA inherently struggles with this relatively simple data set since the decision boundary of an OPLS-DA model will always have a common center-point, due to its “one-vs-rest” setup. This causes trouble if we have more than two classes and the classes are separated along the same variables but at different scales. This is the case for the Iris data set. When fitting an OPLS-DA model to a simplified version of this data set the common center-point results in a suboptimal decision boundary (Figure 2a). OPLS-HDA handles the placement of decision boundaries in a much better way (Figure 2b). By first calculating the distances between the three classes and performing HCA, we create a dendrogram (Figure 2c), that acts as the foundation for the OPLS-HDA model. We then create OPLS-DA models in split #1 and #2 (Figure 2d,e). The dendrogram complete with the two two-class models creates the decision tree and makes up the OPLS-HDA model. The resulting decision boundary of the OPLS-HDA model is a combination of the models in split #1 and #2 shown in Figure 2b.

## Software

For the OPLS-DA, we have utilized SIMCA, version 18.0.0 (Sartorius Stedim Data Analytics, Umeå, Sweden), as the backbone for model building including the cross-validation scheme. In HCA, the hierarchy can be created by linking observations differently by choosing a specific linkage algorithm. Throughout this work, we have applied SciPy to perform the HCA calculations with existing linkage choices.^18^ The benchmarks presented in Supporting Information are performed in python using Scikit-learn^19^ and eigenvector for AHiMBu.^12,13^

## Data Sets

### Glioma Subtypes—Metabolomics

The brain tumor metabolomics data includes 232 observations and 240 identified metabolites originating from cross-platform gas chromatography-mass spectrometry (GC-MS) and liquid chromatography with tandem mass spectrometry (LC-MS/MS) global metabolomics analysis.^8^ The observations were subclassified based on molecular classifications according to WHO 2016 classification of tumors of the central nervous system. According to this molecular and histopathological classification, the material is distributed over seven adult glioma subtypes: 1. glioblastoma, IDH-wildtype; 2. glioblastoma, IDH mutant; 3. astrocytoma, IDH-wildtype; 4. astrocytoma, IDH mutant; 5. oligodendroglioma, IDH mutant and 1*p*/19q-codeleted; 6. oligodendroglioma, NEC (IDH-wildtype and 1*p*/19q-codeleted); and 7. gliosarcoma, IDH-wildtype.

#### Whitefish—NIR Spectra

The Whitefish data set includes 1,311 observations and 125 variables originating from near-infrared (NIR) Spectral data. The data set contains 12 classes consisting of 4 fish species in 3 different states; frozen, fresh or thawed providing two main sources of class variation. This data set is a robust use case for evaluating the efficacy of classification in scenarios where the variable space is correlated with a complex class structure. While the full data set included 18 classes, we decided to restrict our study to a subset of 12 classes including species that had all three states measured.^20^

## Results

### Metabolomics Glioma—Streamlined Identification of Distinct Metabolic Characteristics

In the previous study,^8^ a manual one-vs-one approach was performed to understand the relationship between the different classes. The findings were then summarized in a dendrogram with HCA and volcano plots were generated based on the clustering. This process involved a lot of manual work, the results of which we can efficiently replicate with OPLS-HDA.

**Figure 3:**
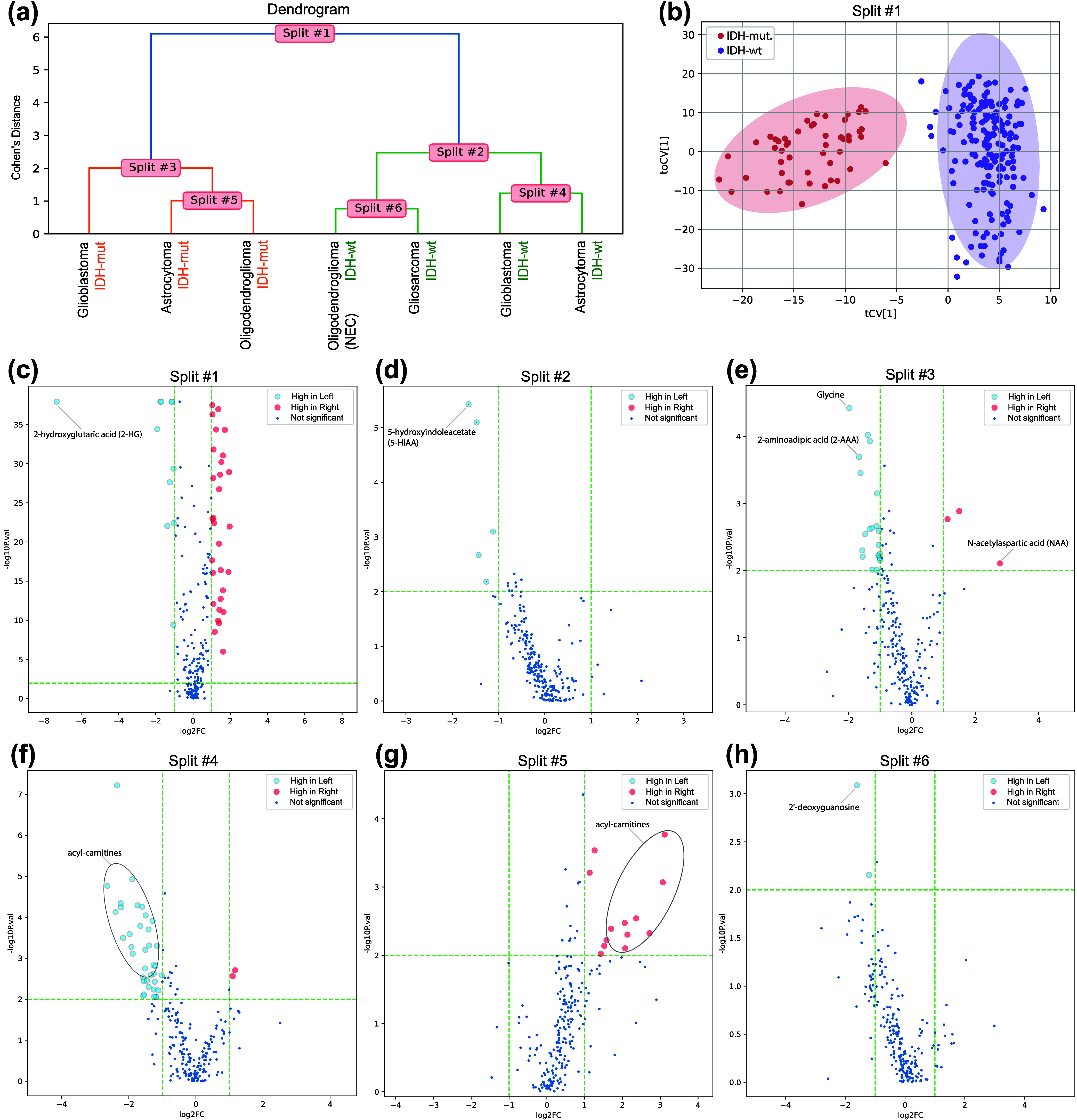
OPLS-HDA results of brain tumor metabolomic data. (a) Dendrogram for the brain tumor metabolomics data of seven WHO defined glioma subtypes. NEC = not elsewhere classified. (b) Cv-score plot for the two-class OPLS-DA model in (a), Split #1, separating IDH wildtype and IDH mutated tumors. (c–h) Volcano plots for each two-class OPLS-DA model in (a), Split #1–6. A few important metabolites, based on statistical significance (*P* < 0.01) and expression level (>2-fold difference), are highlighted. Metabolite fold differences are shown as ratios according to the analyzed Split, as illustrated in the dendrogram in (a). I.e “High in Left” or “High in Right” refers to left- or right-hand side of the Split shown in (a).

To illustrate this, we applied OPLS-HDA to the high-dimensional mass spectrometry-based metabolomics data from the brain tumor tissues. The data included seven tumor subtypes, based on the WHO classification for tumors of the nervous system, including both molecular and histopathological analysis.^21^ Using tumor metabolic phenotypes as input, OPLS-HDA groups the seven glioma subtypes according to their WHO classification in a dendrogram (Figure 3a). The dendrogram, uses Cohen’s *d* on the *y*-axis to reflect class proximity, providing an intuitive understanding of the relationships between the classes. The way OPLS-HDA visualize the data expedites initial data analysis, making it particularly useful of understanding major differences as well as unique features of defined classes in complex data, such as clinical omics data. Split #1 reveals a pronounced distinction between isocitrate dehydrogenase wild type (IDH-wt) and mutated tumors. This separation can also be visualized through traditional cv-score plots from the two-class OPLS-DA model (Figure 3b).

To further investigate the unique features driving the separation of the different tumor classes, we generate volcano plots for each two-class OPLS-DA model, based on statistical significance and expression level for individual metabolites (Figure 3c–h). This analysis shows that Split #1, which separates the three IDH-mutated classes from the four IDH-wt classes, is driven by 41 metabolites with significantly different levels in the tissues (*P* < 0.01 and >2-fold difference). The most important metabolite is the high expression of 2-hydroxyglutaric acid (2-HG) produced in IDH-mutated cells (Figure 3c).

Focusing on the subsequent splits, we see that Split #2 differentiates IDH-wt classes; glioblastoma and astrocytoma from oligodendroglioma (NEC) and gliosarcoma. This differentiation is primarily driven by high levels of 5-hydroxyindoleacetate, a metabolite of serotonin catabolism (Figure 3d).^8^ Turning our attention to the IDH-mutated classes, Split #3 distinguishes high-grade glioblastoma from lower-grade astrocytoma and oligodendroglioma tumors. Metabolic markers for rapid cell proliferation, such as glycine and 2-aminoadipic acid, were prominent in high-grade glioblastoma while *N*-acetylaspartic acid, a metabolic marker for normal nervous tissue, was low as expected (Figure 3e). As we move down the dendrogram, the differences between analyzed classes becomes less pronounced, which is anticipated as the tumors metabolic phenotypes becomes more similar. However, both Split #4 and Split #5, regardless of IDH mutation status, show reduced levels of a broad range of acyl-carnitines in lower-grade astrocytoma tumors, highlighting these metabolites as unique features (Figure 3f,g).

### Whitefish Data Set—Hierarchy and Score Plots

**Figure 4:**
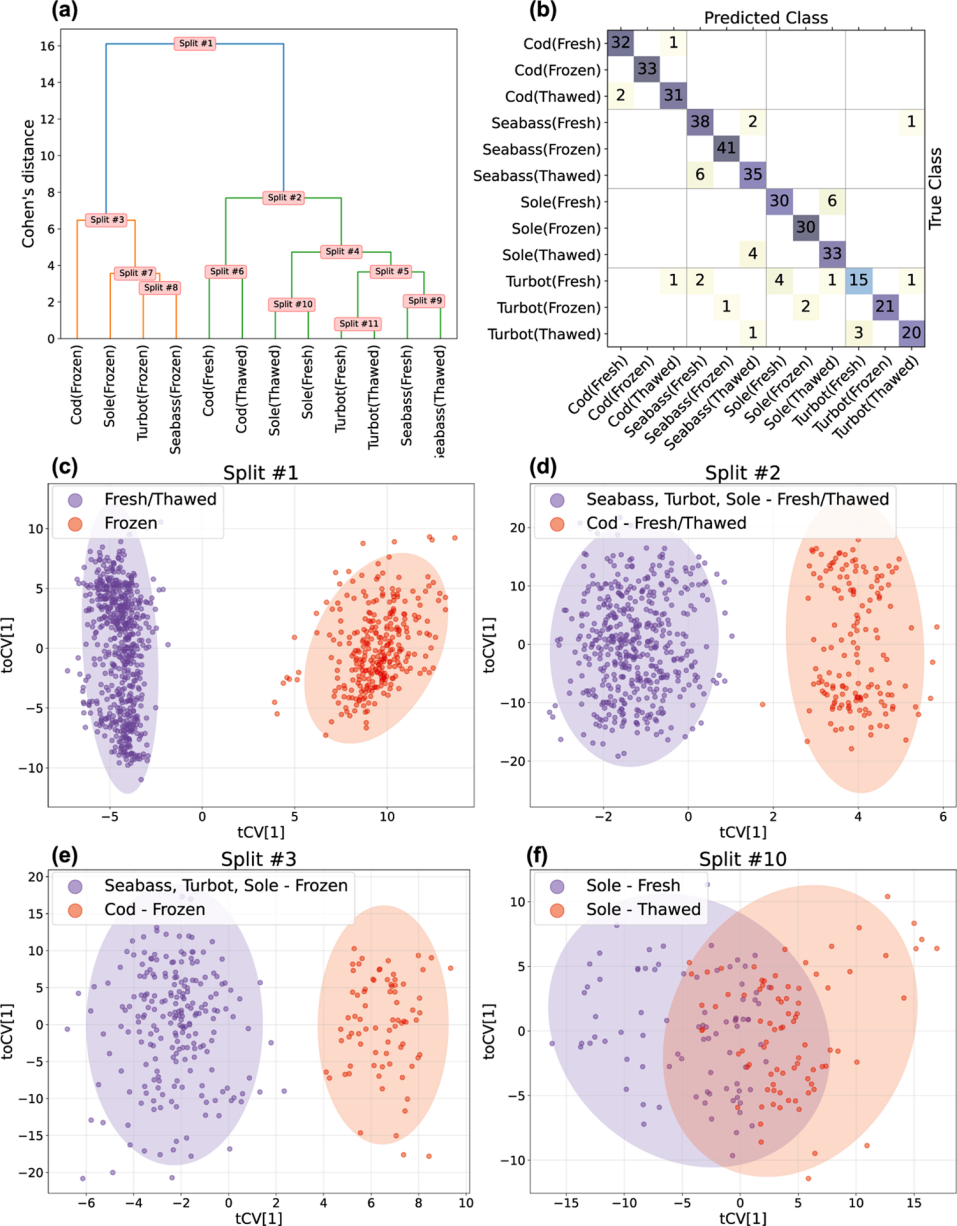
(a) Dendrogram of the whitefish data produced by the Cohen’s *d* calculated from the ***ŷ***_cv_ of 66 one-vs-one OPLS-DA models. Frozen classes are separated from fresh/thawed and cod is separated from the other species. (b) Confusion matrix of the test set with an accuracy of 90.4% with the actual class belongings on the *y*-axis and the predicted on the *x*-axis. (c–f) Cross-validated score plots from two-class OPLS-DA models in splits #1, #2, #3 and #10, from the OPLS-HDA model shown in (a). Split #1–3(c–e) are clearly separated while #10 has a significant overlap. The ellipses around each class in the score-plots (c–f) are 95% confidence intervals for each class based upon Hotelling’s T2.

In the Whitefish study, 66 one-vs-one OPLS-DA models are summarized and presented in a dendrogram, as shown in Figure 4a. This dendrogram provides clear insights of how different groups relate to each other, namely that the state of the fish is the main source of variation and thus frozen samples differ significantly from fresh or thawed ones. Speculatively, this is because of the presence of ice in the frozen samples. Next, we see that the same species that are either fresh or thawed are more similar to each other and that cod stands out distinctly from other species, regardless of whether the samples are frozen or fresh/thawed.

These observations are supported by the cv-score plots from the OPLS-DA models in the splits, shown in Figure 4c–f. Similarly, the confusion matrix in Figure 4b confirms these findings, i.e., as we move down the tree, the likelihood of correctly classifying a sample decreases. Notably, no frozen sample was misclassified as fresh or thawed. Also, cod is consistently classified separately from other species. The primary challenge involves confusing fresh and thawed samples within the same species, as illustrated in Figure 4f, where the cross-validated scores for fresh and thawed sole overlap significantly.

### Benchmark Results

To compare OPLS-HDA with other multiclass classification methods, we have performed a benchmark on a wide range of data sets. The data sets range from simple ones as Iris^17^ to high-dimensional once such as the Breast Cancer data set.^22^ We compare the performance of OPLS-HDA to eight other methods (Table 1). From this, OPLS-HDA emerges as a strong and flexible competitor to the other methods. Being in the top three for all data sets except LIVECell results in OPLS-HDA having the highest average accuracy of all the tested methods. This displays the robustness and flexibility of OPLS-HDA to handle both low- and high-dimensional data sets with varying characteristics. A more in-depth description of the data sets and the tested methods are presented in Supporting Information.

**Table 1:**
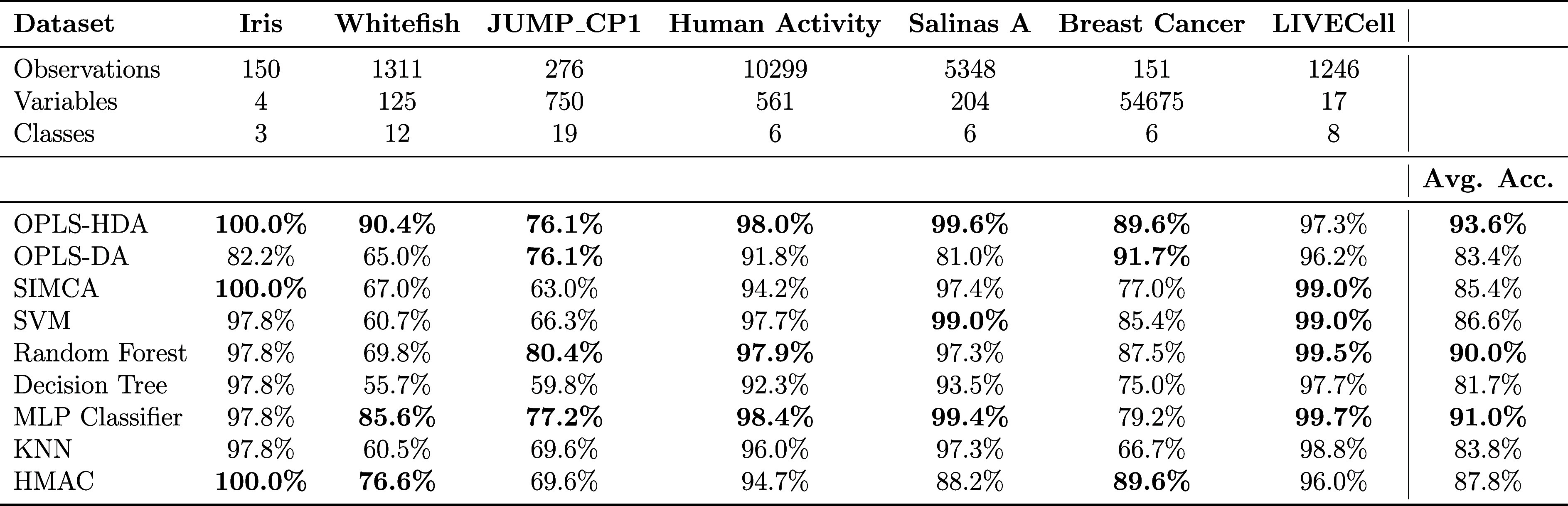
Combined Dataset Summary and Benchmarking Results^a^

a The top-three methods are highlighted in bold.

## Conclusions

In this paper, we have introduced a new method for multiclass classification called orthogonal partial least squares-hierarchical discriminant analysis (OPLS-HDA). This method is based on OPLS, a widely used chemometric tool with applications in both industry and academia. The motivation behind this method is that OPLS-Discriminant Analysis (DA), which can be used for multiclass classification, often struggles as the number of classes increases. For two-class classification, OPLS-DA is highly effective, offering strong discrimination between classes and providing interpretable information on why the two classes differ. OPLS-HDA integrates the strengths of two-class OPLS-DA models with HCA to create a top-down OPLS-DA-based decision tree.

In the glioma study, we showed that OPLS-HDA results in much less manual work but with the same interpretation as the original paper.^8^ The whitefish NIR data set differs in both state and species where OPLS-HDA clearly separated the biggest source of variation to smaller sources. Further, we have benchmarked OPLS-HDA on eight data sets and compared the classification test set accuracy to eight popular machine learning models.

We demonstrate that with OPLS-HDA’s data-driven decision-making, we avoid tedious manual work while still allowing for a detailed interpretation of the difference between classes. This approach allows researchers to focus on interpreting their data and find the answers to their research questions instead of a time-consuming model-building process. This methodology not only streamlines the analytical process but also enhances the robustness and clarity of the results, paving the way for more efficient and insightful multiclass research in the future.

## Data Availability

All code and data can be reached at: https://github.com/edvinforsgren/OPLS-HDA/, except for the glioma study data, which can be shared upon reasonable request to the authors. Publicly sharing of clinical data is not permitted according to written informed consent.

## Supporting Information Available

Benchmarking; method descriptions: soft independent modeling of class analogy—SIMCA;^23−25^ automatic hierarchical classification model builder (AHIMBU)—hierarchical model automatic classifier (HMAC);^12,13^ k-nearest neighbor;^26,27^ decision tree;^28^ random forest;^29^ suport vector machines;^30^ multi-layer perceptron (MLP)—artificial neural network (ANN);^31^ datasets; Iris;^17^ subset of CP1_JUMP; human activity;^32^ Salinas A;^33^ breast cancer;^22^ LIVECell—cell morphology^34^ (PDF)
