# Supplementary material for: OPLS-Based Multiclass Classification and Data-Driven Interclass Relationship Discovery
Source: J Chem Inf Model. 2025 Feb 3;65(4):1762–70. doi: 10.1021/acs.jcim.4c01799 (PMC11863383; doi:10.1021/acs.jcim.4c01799)
Supplement: Supplementary file 1 — ci4c01799_si_001.pdf [file ci4c01799_si_001.pdf]

Supplementary Information for: OPLS-based Multiclass Classification and Data-Driven Inter-Class Relationship Discovery

Edvin Forsgren,*,† Benny Björkblom,‡ Johan Trygg,†,¶ and Pär Jonsson¶

†*Computational Life Science Cluster (CLiC), Department of Chemistry, Umeå University, SE-901 87, Umeå, Sweden*

‡*Department of Chemistry, Umeå University, SE-901 87, Umeå, Sweden*

¶*Sartorius Corporate Research, SE-903 33, Umeå, Sweden*

E-mail: edvin.forsgren@umu.se

Benchmarking

To ensure OPLS-HDA is a robust multiclass alternative, we have performed a benchmark on a wide range of datasets. The datasets range from simple ones as Iris with 3 classes and 4 variables to truly high-dimensional once with tens of thousands of variables with a lot fewer observations. We compare the performance of OPLS-HDA to 7 other established methods.

Method descriptions

Soft Independent Modeling of Class Analogy - SIMCA

Soft independent modeling of class analogy (SIMCA) is a multivariate method where one PCA model is created for each class. To classify an observation, the residual, Distance to model in X-space (DmodX), is used to see if the observations fit into the PCA model or not.

SIMCA is often used in process control to make sure that a process is not deviating from historical data. It is effective in these circumstances, but is a lot harder to interpret than OPLS-DA.[1–3]

Automatic Hierarchical Classification Model Builder (AHIMBU) - Hierarchical Model Automatic Classifier (HMAC)

Automatic Hierarchical Classification Model Builder (AHIMBU) or Hierarchical Model Automatic Classifier (HMAC) as the method is called in Eigenvectors implementation. AHIMBU is a method that tries to solve the same problems as OPLS-HDA with PLS-DA models. Similarly to OPLS-HDA, AHIMBU begins with creating one-vs-one models to map the relationship between classes. But then, the two classes that have the lowest success rate (highest classification error) are selected and merged into one class. The minimum cross-validated nonerror rate is used to estimate the classification error. If all classes then can be separated perfectly in one model, or if there are only two classes remaining, all the relevant models are built. If this is not the case, then the process is repeated. This will, similarly to OPLS-HDA, result in a decision tree based on PLS-DA models. However, OPLS-HDA can handle orthogonal variation, and utilizes Hierarchical Cluster Analysis (HCA) based on a distance matrix of statistical measures of effect (Cohen's distance) to achieve its structure whereas AHIMBU relies solely on classification error.[4,5] The difference becomes clear when the two methods are benchmarked on several datasets.

k-Nearest Neighbor

The k-Nearest Neighbors (k-NN) algorithm is a non-parametric method meaning that there is actually no "model" that needs to be trained or tuned. It instead classifies a data point by analyzing the 'k' closest labeled data points and adopting the most common class among them, based on a distance metric. The distance calculations for prediction are computationally intensive with big datasets but often works well as long as the space is not too

high-dimensional.[6,7]

Decision Tree

The Decision Tree is a predictive method used in statistics, data mining, and machine learning. It uses a tree-like model of decisions and their possible consequences. Decision Trees are simple to understand and interpret, but they are prone to overfitting, and often perform poorly on test data.[8]

Random Forest

Random Forest is an ensemble learning technique constructed of multiple decision trees. The prediction is then based on a majority voting of the decision trees in the forest. This method addresses one of the key limitations of a single decision tree, which is the tendency to overfit the training data. A Random Forest can handle large datasets efficiently and is used in diverse applications such as credit scoring and disease prediction. However, for complex data, the understanding and interpretation of the decisions and predictions are challenging.[9]

Suport Vector Machines

Support Vector Machines (SVM) is a popular method for classification that is especially efficient in high-dimensional spaces. SVM works by constructing a hyperplane or a set of hyperplanes in a high-dimensional space. This space can then be used for classification or regression. In classification, the core idea is to maximize the margin between the data points of different classes, effectively creating the widest possible gap between classes. However, tuning hyperparameters can be challenging to find an appropriate fit. Interpreting the decision boundary can be done in lower dimensions, but with growing dimensions the interpretability becomes challenging.[10]

Multi-Layer Perceptron (MLP) - Artificial Neural Network (ANN)

Multi-Layer Perceptron (MLP) is a class of feedforward artificial neural network (ANN). An MLP consists of at least three layers of nodes: an input layer, a hidden layer, and an output layer. Except for the input nodes, each node is a neuron that uses a nonlinear activation function. MLP utilizes backpropagation to optimize the model. MLPs are widely applied in many fields but require large datasets and careful tuning of hyperparameters. As widely recognized, the interpretation of ANNs is hard.[11]

Each of these methods has its strengths and weaknesses, making them suitable for different types of problems in the field of machine learning. The choice of algorithm often depends on the size of the dataset, the nature of the problem, the computational resources available, and how valued interpretation.

Datasets

The datasets were chosen to represent a wide range of different data types, number of classes, observations, and variables. This, to ensure OPLS-HDA is not only applicable on certain datatypes but performs well in most settings.

Iris

The Iris dataset spanns 150 observations across 3 classes. The variables are the lengths and widths of the sepals and petals of three species of iris plants. Although with only 4 variables, this dataset is widely used as a comprehensive benchmark example.[12]

Subset of CP1_JUMP

The subset of CP1_JUMP dataset, comprising 276 observations and 750 variables derived from CellProfiler features, and spans 19 classes of compound treatments. In this high-

dimensional dataset, the variable space outweighs the observational space. It offers a challenging task for methods to effectively discern between numerous classes, all while combating the challenges posed by the curse of dimensionality.

Human Activity

The Human Activity dataset houses data from 10,299 observations, each described through 561 variables, and consists of 6 activity classes. The variables are drawn from cell phone accelerometers and describes patterns and trends in human motions.[13]

Salinas A

In the domain of remote sensing, the Salinas A dataset, with 5,348 observations and 204 variables spread across 6 classes, is derived from hyperspectral images of land. The spectral data, challenges the methods with highly correlated variables.[14]

Breast Cancer

The Breast Cancer dataset, hosting data from 151 observations across 6 classes, is characterized by a variable space of 54,675 gene expression variables. It is a truly high-dimensional biological data scenario, with a very limited number of observations.[15]

LIVECell - Cell Morphology

The LICVECell dataset is a collection of phase-contrast images of cells from 8 different cell lines imaged at different time points.[16] The cells are grown in wells and each well is imaged at two regions of interest (ROI). 17 image features (variables) describing each cell's morphology have been extracted using single-cell data.For each region, the median of all cells is used, resulting in 1246 observations, split into a training and a test set. Each well could only be part of either the training or the test set to reduce overfitting and maximize the variability between the two sets of data.

These public datasets, each with their unique challenges and characteristics, provide a comprehensive and diverse array to validate the OPLS-HDA methodology across various domains, dimensionalities, and class structures. Consequently, they offer a wide view of its performance and applicability in varied real-world scenarios, ranging from simple and direct classifications to high-dimensional, multiclass situations.

References

(1) Wold, S. Pattern recognition by means of disjoint principal components models. *Pattern Recognition* **1976**, *8*, 127–139.

(2) Bicciato, S.; Luchini, A.; Di Bello, C. Marker identification and classification of cancer types using gene expression data and SIMCA. *Methods of Information in Medicine* **2004**, *43*, 4–8.

(3) Wold, S.; Sjöström, M. SIMCA: A Method for Analyzing Chemical Data in Terms of Similarity and Analogy. Chemometrics Theory and Application. 1977; pp 243–282.

(4) Marchi, L.; Krylov, I.; Roginski, R. T.; Wise, B.; Di Donato, F.; Nieto-Ortega, S.; Pereira, J. F. Q.; Bro, R. Automatic hierarchical model builder. *Journal of Chemometrics* **2022**, *36*, e3455.

(5) HMAC - Eigenvector Research Documentation Wiki. `https://wiki.eigenvector.com/index.php?title=Hmac`, Accessed: 2024-04-12.

(6) Fix, E.; Hodges, J. L. 1951, DOI: `10.1037/e471672008-001`, PsycEXTRA Dataset.

(7) Cover, T.; Hart, P. Nearest neighbor pattern classification. *IEEE Transactions on Information Theory* **1967**, *13*, 21–27.

(8) Quinlan, J. R. Induction of decision trees. *Machine Learning* **1986**, *1*, 81–106.

(9) Breiman, L. Random forests. *Machine Learning* **2001**, *45*, 5–32.

(10) Cortes, C.; Vapnik, V. Support-vector networks. *Machine Learning* **1995**, *20*, 273–297.

(11) Rumelhart, D. E.; Hinton, G. E.; Williams, R. J. Learning representations by back-propagating errors. *Nature* **1986**, *323*, 533–536.

(12) Fisher, R. A. The use of multiple measurements in taxonomic problems. 1936.

(13) Anguita, D.; Ghio, A.; Oneto, L.; Parra, X.; Reyes-Ortiz, J. L. Human Activity Recognition Using Smartphones Dataset. UCI Machine Learning Repository, 2012; `http://archive.ics.uci.edu/ml/datasets/Human+Activity+Recognition+Using+Smartphones`.

(14) Group of Computational Intelligence, U. o. t. B. C. Salinas-A Scene Hyperspectral Dataset. Hyperspectral Remote Sensing Scenes, 2023; `https://www.ehu.eus/ccwintco/index.php/Hyperspectral_Remote_Sensing_Scenes`, Accessed: [insert today's date].

(15) Network, T. C. G. A. Gene expression dataset of breast cancer biopsy samples. NCBI GEO, 2005; `https://www.ncbi.nlm.nih.gov/geo/query/acc.cgi?acc=GSE1456`.

(16) Edlund, C.; Jackson, T. R.; Khalid, N.; Bevan, N.; Dale, T.; Dengel, A.; Ahmed, S.; Trygg, J.; Sjögren, R. LIVECell—A large-scale dataset for label-free live cell segmentation. 2021.
